# Antimicrobial Resistant *Staphylococcus* spp., *Escherichia coli*, and *Salmonella* spp. in Food Handlers: A Global Review of Persistence, Transmission, and Mitigation Challenges

**DOI:** 10.3390/pathogens14050496

**Published:** 2025-05-18

**Authors:** Gustavo Guimarães Fernandes Viana, Marita Vedovelli Cardozo, Juliano Gonçalves Pereira, Gabriel Augusto Marques Rossi

**Affiliations:** 1 Department of Animal Production and Preventive Veterinary Medicine, School of Veterinary Medicine and Animal Science, São Paulo State University (UNESP), Botucatu 18618-681, SP, Brazil; gustavo.zero98@gmail.com (G.G.F.V.); juliano.pereira@unesp.br (J.G.P.); 2 Department of Pathology, Reproduction and One Health, Faculty of Agricultural and Veterinary Sciences, São Paulo State University (UNESP), Jaboticabal 14884-900, SP, Brazil; marita.vedovelli@unesp.br; 3 Department of Veterinary Medicine, University Vila Velha (UVV), Av. Comissário José Dantas de Melo, n.21, Vila Velha 29102-920, ES, Brazil

**Keywords:** antibiotic resistance, foodborne pathogens, food safety, hygiene practices, one health

## Abstract

Antimicrobial resistance in foodborne pathogens represents a critical global health challenge, with food handlers serving as key contributors in their transmission. This comprehensive review synthesizes evidence on the prevalence, transmission dynamics, and antimicrobial resistance patterns of three major pathogens, *Staphylococcus* spp., *Escherichia coli*, and *Salmonella* spp., among food handlers worldwide. Analysis of studies across diverse geographical regions reveals considerable variation in colonization rates, with *Staphylococcus* spp. prevalence ranging from 19.5% to 95.0%, *Escherichia coli* from 2.8% to 89.3%, and *Salmonella* spp. from 0.07% to 9.1%. Resistance profiles demonstrate alarming trends, including widespread β-lactam resistance and emerging resistance to last-resort antibiotics like carbapenems. Particularly concerning is the high occurrence of multidrug resistant (MDR) strains and extended spectrum β-lactamase (ESBL) producers in low- and middle-income countries. This review identified inadequate handwashing, poor hygiene infrastructure, and asymptomatic carriage as critical factors facilitating the transmission of antimicrobial resistant strains. These findings underscore the urgent need for enhanced surveillance systems, targeted decolonization strategies, improved hygiene protocols, and food handler education to mitigate the spread of resistant pathogens through the food chain.

## 1. Introduction

Antimicrobial resistance (AMR) is a critical global health challenge, posing as a major threat to modern medicine and public health [1]. It occurs when microorganisms such as bacteria, viruses, fungi, and parasites evolve to resist the effects of antimicrobial agents, rendering standard treatments ineffective [2]. AMR has escalated dramatically over the past decade, with bacterial AMR directly causing 1.27 million deaths globally in 2019 and contributing to nearly 5 million deaths annually [3]. Over the past decade, the detection of multidrug resistant pathogens has surged, particularly in low- and middle-income countries (LMICs), where limited healthcare infrastructure and unregulated antibiotic use amplify risks [4,5]. Projections suggest that by 2050, AMR could result in up to 8 million deaths per year, accompanied by substantial economic losses estimated at $1 trillion to $3.4 trillion annually in GDP [6,7]. The misuse and overuse of antimicrobials in human medicine, agriculture, and animal production are primary drivers of this crisis [8]. This crisis is compounded by the globalization of food trade, which facilitates the spread of resistant strains across borders, turning localized outbreaks into international emergencies [9,10].

**Figure 1:**
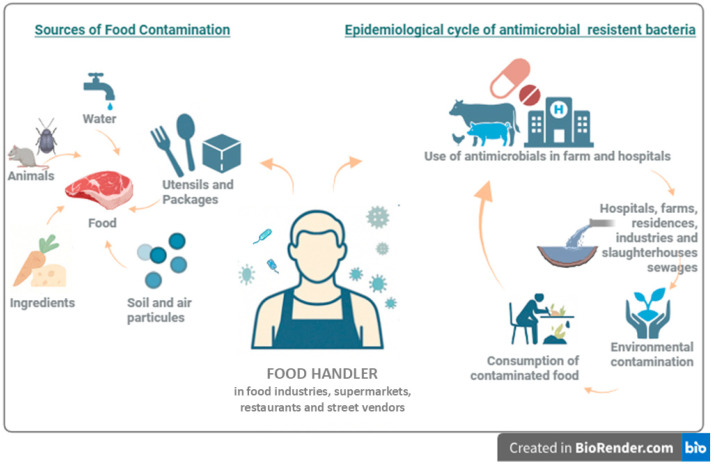
The image illustrates the role of the food handlers within two distinct yet interconnected cycles. On the left, the food handler is depicted as a potential source of food contamination, alongside other contributors such as animals, raw ingredients, water, airborne and soil particles, utensils, and packaging materials. On the right, the diagram highlights the food handler’s involvement in the epidemiological cycle of antimicrobial-resistant bacteria dissemination. This cycle encompasses several interacting factors, including the use of antimicrobials in agricultural and healthcare settings, which contributes to the production of contaminated sewage originating from hospitals, slaughterhouses, households, and industrial facilities. Such waste leads to environmental contamination—affecting water sources, soil, animals and crops—which may, in turn, result in the consumption of contaminated food products. Critically, the food handler plays an active role in this dynamic when working in restaurants, street food stalls, or similar environments, where improper handling practices may lead to the contamination of various types of food. When such foods are consumed raw or are insufficiently cooked, the contamination can reach the consumer directly, thereby contributing to the transmission of pathogenic and antimicrobial-resistant bacteria.

Among the various contributors to AMR dissemination, food handlers, including workers involved in food processing, preparation, and serving, play a critical role in persistence and spreading antimicrobial resistant bacteria through improper hygiene practices and food handling behaviors (Figure 1) [11].

Food handlers may serve as carriers of resistant bacteria including *Escherichia coli*, *Staphylococcus* spp. (mainly *S. aureus*), and *Salmonella* spp., all of which are frequently implicated in foodborne illnesses worldwide [12,13,14,15]. These pathogens are often resistant to critically important antibiotics like ciprofloxacin, carbapenems, and third-generation cephalosporins [16,17,18]. Contamination can occur through direct contact with food or surfaces due to poor hand hygiene, sneezing, or coughing near food, handling raw and cooked foods without proper sanitation, or neglecting personal protective measures like gloves [19,20,21]. Critically, sublethal exposure to antimicrobials during food processing can promote resistance gene transfer among bacteria, as stressed cells may undergo horizontal gene transfer. Mobile genetic elements like plasmids, integrons, and transposons enable the rapid exchange of resistance genes, allowing commensal bacteria to transfer resistance traits to pathogenic strains within food matrices [22].

The transmission of resistant bacteria by food handlers is exacerbated by inadequate training in Good Manufacturing Practices (GMP), lack of awareness about food safety protocols, and unsanitary behaviors such as failing to wash hands after using the restroom or touching contaminated surfaces. Studies have consistently shown a strong correlation between poor hygiene practices among food handlers and the presence of resistant bacteria on their hands or in prepared foods [23,24,25,26]. The persistence of resistant strains is further exacerbated in environments with inadequate sanitation, where biofilms and persister cells can evade standard disinfection protocols [27].

Addressing AMR in the food production chains demands a holistic approach, with food handlers serving as a critical intervention point. As gatekeepers of food safety, their practices directly influence whether resistant pathogens reach consumers, making their role indispensable in global AMR mitigation efforts [28,29].

The aim of this study is to review the prevalence, transmission dynamics, and antimicrobial resistance patterns of three major bacterial groups frequently associated with foodborne transmission—*Staphylococcus* spp., *Escherichia coli*, and *Salmonella* spp.—among food handlers across diverse geographical regions. While *S. aureus* and specific pathogenic *E. coli* strains are widely recognized as foodborne pathogens, this review includes all *Staphylococcus* and *E. coli* isolates reported in the literature to ensure a comprehensive synthesis of available data. The review also aims to identify key factors that contribute to the spread of resistant strains and propose strategies to mitigate this issue.

## 2. Methodology

This descriptive literature review was conducted to gather and analyze studies reporting on the presence of *Escherichia coli*, *Salmonella* spp., and *Staphylococcus* spp. in food handlers, with a particular focus on AMR. The search was carried out between February and May 2025 using three electronic databases: Google Scholar, ScienceDirect, and PubMed. Articles published within the last 25 years were considered eligible for inclusion, with priority given to more recent publications to ensure the relevance and timeliness of the information. Studies were selected based on their focus on food handlers as sources of bacterial contamination and the presence of antimicrobial resistance patterns, AMR genes, or multidrug resistant isolates. The selected literature includes peer-reviewed research articles that provided either phenotypic or genotypic data on antimicrobial resistance. Although a wide range of search terms were used during the data collection phase, including combinations of terms such as “antimicrobial resistance”, “food handlers”, “ESBL”, “MRSA”, “*Escherichia coli*”, “*Salmonella*”, and “*Staphylococcus*”, these are not exhaustively listed here to maintain readability.

## 3. *Staphylococcus* spp.

### 3.1. Overview of Staphylococcus Genus

*Staphylococcus* spp. is a Gram-positive, facultative anaerobic bacterium that asymptomatically colonizes the nasal passages, skin, and mucosal surfaces of approximately 30.0% of the global population [30,31]. Most symptoms from *Staphylococcus aureus* food poisoning range from nausea, vomiting, and abdominal cramping with or without diarrhea to life-threating infections in vulnerable populations and depending on the amount of enterotoxins ingested. Usually, the most common symptoms occur 30 min to 8 h after ingestion [32,33].

While many strains are harmless, pathogenic *S. aureus*, the most prevalent species of the genus *Staphylococcus*, produces virulence factors such as cytotoxins, adhesins, and superantigens, including staphylococcal enterotoxins (SEs) and toxic shock syndrome toxin-1 (TSST-1) [12,34]. Methicillin-resistant *Staphylococcus* (MRSA), a multidrug-resistant variant, has evolved resistance to β-lactams and other antimicrobial classes through the acquisition of the *mecA* gene, which encodes penicillin-binding protein 2a (PBP2a) [35,36]. *Staphylococcus* spp. is a leading cause of healthcare and community acquired infections, but its role in foodborne outbreaks underscores its adaptability and public health importance. The bacterium is most prevalent in foods like dairy, meats, and ready-to-eat salads, making food handlers critical for contamination through improper hygiene practices [37,38,39].

### 3.2. Staphylococcus aureus as a Common Foodborne Pathogen

*S. aureus* is a major foodborne pathogen due to its ability to produce heat-stable enterotoxins (SEs) that withstand cooking temperatures [40,41]. These toxins, encoded by genes such as *sea*, *seb*, *sec*, *sed*, and *see*, are preformed in food and cause rapid-onset illness, with even low toxin concentrations of SEs being sufficient to cause food poisoning [42,43]. The ubiquity of *S. aureus* in human carriers, particularly food handlers, facilitates cross-contamination [29]. Persistent colonization, often asymptomatic, allows continuous contamination of food during preparation, especially in settings with inadequate hygiene protocols [44]. Beyond foodborne intoxications, *S. aureus* is also recognized as a leading cause of both hospital- and community-acquired infections [45]. The widespread use of antibiotics to treat these infections has substantially contributed to the rise of antimicrobial resistance in this species, complicating treatment and increasing the risk of therapeutic failure [46].

### 3.3. Colonization and Transmission Dynamics Related to Food Handlers

Persistent nasal carriers play a critical role in environmental contamination. In environments with inadequate disinfection, *Staphylococcus* spp. from colonized handlers can colonize shared surfaces, utensils, or equipment [47,48]. A study demonstrated that 54.7% of hand contamination events in non-colonized workers were linked to strains from persistently colonized coworkers, highlighting the role of indirect transmission via shared workspaces [49]. This pathway is particularly important in settings where high-touch surfaces (e.g., countertops, knives) are not routinely sanitized [50]. Biofilm formation on skin and environmental surfaces further enhances persistence, complicating eradication efforts in food processing environments [51].

Handlers may acquire *Staphylococcus* spp. through contact with raw or undercooked animal products [52]. *S. aureus* strains recovered from foods and food handlers in Spain shared similar clonal complexes, carried important virulence genes, and exhibited antimicrobial resistance [53]. For instance, spa type t127, commonly associated with livestock, was prevalent among handlers in a study by Baptistão [54], indicating that handling contaminated meat or dairy products may introduce animal-derived strains into human carriers.

Asymptomatic carriers, particularly those with intermittent or persistent nasal colonization, are key sources of contamination [47]. Food handlers may carry *Staphylococcus* spp. exclusively on their hands (without nasal colonization), likely due to contact with contaminated surfaces rather than self-inoculation. This suggests that even handlers without nasal colonization can transmit the pathogen if hygiene protocols are breached [55].

### 3.4. Antimicrobial Resistance in Staphylococcus spp. Isolated from Food Handlers

Table 1 represents the major results from most of the studies utilized in this review for antimicrobial-resistant *Staphylococcus* spp. isolated from food handlers worldwide.

The prevalence of *Staphylococcus* spp. among food handlers represents a major public health concern globally, particularly due to the emergence of antimicrobial-resistant strains. This review synthesizes findings from multiple international studies, revealing substantial variations in colonization rates, resistance patterns, and genetic determinants across different geographical regions and sample types. Understanding these patterns is essential for developing effective infection control strategies in food service environments, aimed at mitigating the risk of foodborne illness transmission.

Two studies evaluated both *S. aureus* and coagulase negative *Staphylococcus*. The study by Adefrash et al. [56] found that coagulase negative *Staphylococci* exhibited a 53.9% prevalence of MDR; similarly, 51.5% of *S. aureus* isolates were MDR. In a study from Morocco, 17.1% of coagulase-negative *Staphylococcus* isolates carried the *mecA* gene, while 43.8% of *S. aureus* isolates were *mecA*-positive; notably, all isolates from this study were classified as MDR [59].

Colonization rates varied widely by geography and sample type, ranging from 12.0% [63] to 95.0% [58]. Sample type analysis revealed important differences in colonization patterns. In one Brazilian study, 29.3% (41/140) of nasal samples compared to 50.0% (70/140) of hand samples were positive for *S. aureus*, indicating higher hand carriage rates [62]. For the study in Morocco, 25 nasal samples (83.3%) and 30 hand samples (75%) were positive for *Staphylococcus* spp. [59]. These variations highlight the importance of considering both geographical factors and methodological approaches when assessing *Staphylococcus* spp. colonization among food handlers.

Hospital-based studies demonstrated higher *Staphylococcus* spp. colonization rates among food handlers (39.6–78.6%) compared to those conducted in other facility types (12.0–33.0%). Interestingly, a Brazilian investigation in a pilot kitchen environment reported an exceptionally high contamination rate of 90.2% [34]. However, this anomalous finding may be attributed to the non-conventional food preparation setting, which likely introduced unique variables that influenced microbial colonization patterns not typically observed in standard food service environments.

The prevalence of methicillin-resistant *S. aureus* among food handlers exhibits substantial geographical heterogeneity. In Iran, 16.5% of *S. aureus* isolates from food handlers were identified as MRSA, contrasting sharply with only 1.0% prevalence of community-acquired MRSA in a control group of healthy volunteers [57]. A Hong Kong research identified 5.0% of *Staphylococcus* isolates as MRSA through detection of the *mecA* gene [66]. Nigerian studies reported MRSA rates of 21.8% in one investigation, while another found 82.1% of isolates resistant to both cefoxitin and oxacillin, suggesting a remarkably high MRSA prevalence [60,65].

Conversely, studies from several regions detected no MRSA isolates. All *S. aureus* strains from Myanmar tested negative for the *mecA* gene [35]. Similarly, Italian research found no MRSA among 28 *S. aureus* isolates from food handlers [44], and Malaysian investigations confirmed 100% of *S. aureus* strains were methicillin susceptible [58].

Egyptian research presented a concerning scenario wherein all isolated *S. aureus* strains (12/40 samples) were phenotypically confirmed as MRSA, and genotypically validated via PCR detection of the *mecA* gene [36]. Additionally, 25.0% of these isolates were identified as vancomycin-resistant *S. aureus* (VRSA), representing a meaningful public health threat. In Morocco, all isolated *Staphylococcus* spp. strains demonstrated resistance to oxacillin, with the *mecA* gene detected in 13 total food handlers’ samples [59].

Molecular characterization of MRSA isolates revealed diverse genetic elements. A Brazilian research identified various SCCmec types among MRSA isolates, including one isolate each of types II, III, IV, and V, with three strains remaining untyped [34]. Analysis of the cassette chromosome recombinase (ccr) gene complex revealed *ccr2* in 85.7% of isolates, while *ccr3* and *ccrC* were present in 28.6%, and exclusively *ccrC* in 14.3% [34]. This genetic diversity suggests multiple evolutionary pathways in the acquisition of methicillin resistance among *S. aureus* strains colonizing food handlers.

Resistance to β-lactam antibiotics was reported across geographical regions. Penicillin resistance was exceptionally high, with studies reporting rates of 83.8% and 93.0% in Brazil [34,62], 92.1% in Nigerian food handlers [60], and 57.9% of isolates demonstrating resistance in one Malaysian research [58]. Italian researchers found 79.0% of isolates resistant to penicillin, with resistance mechanisms associated with the presence of the *blaZ* gene [44]. Moroccan studies reported 100% resistance to penicillin among all *S. aureus* isolates from both nasal and hand samples [59].

Oxacillin resistance, indicative of MRSA, showed greater geographical variation. A Brazilian research documented 59.5% resistance [34], while a different study in the country reported 50.0% resistance [62], and a Nigerian investigation found 82.1% of isolates resistant to oxacillin [65]. In stark contrast, all isolates from Myanmar were susceptible to oxacillin [35]. Cefoxitin resistance, another MRSA marker, ranged from 8.11% [34] to 57.7% [62] in different Brazilian studies and 82.1% in Nigerian food handlers [65].

Vancomycin resistance, particularly concerning due to its role as a last-resort antibiotic, was observed at alarming rates in certain regions. While one Brazilian study reported no vancomycin resistance [34], 72.9% of isolates in another investigation in the country exhibited resistance [62]. The Nigerian studies included in this review ranged from 0.0% to 3.9% resistance [60,65].

Linezolid resistance was relatively uncommon, with a Brazilian and Hong Kong study reporting no resistance [34,66] and a Nigerian investigation showing 85.7% susceptibility [65]. Paradoxically, Italian research documented 68.0% of isolates resistant to linezolid, an unusually high rate requiring further investigation [44].

Macrolide resistance was highly prevalent across multiple regions. Erythromycin resistance was identified in 67.6% and 52.8% of Brazilian isolates [34,62], 50.0% in Nigerian food handlers [65], and 16.0% in another investigation in Hong Kong [66]. Italian research found 32.0% of isolates resistant to erythromycin, associated with the presence of the *msrA* gene [44]. In Malaysia, 13.7% of isolates demonstrated erythromycin resistance [58].

Clindamycin resistance showed greater regional variation, with rates of 2.7% in Brazilian isolates [34] and 46.4% in Nigerian food handlers [65]. An Italian research identified only one strain with clindamycin resistance, linked to the presence of the *linA* gene [44]. A Malaysian study reported 3.2% resistance to clindamycin [58].

Fluoroquinolone resistance varied considerably by region. Ciprofloxacin resistance ranged from 0.0% in one study [61] to 32.8% in another [62], 50.0% in Moroccan nasal isolates [59], and 7.0% in a separate investigation [66]. Nigerian food handlers demonstrated relatively high ciprofloxacin susceptibility (40.6% and 67.9%) [60,65].

Aminoglycoside resistance was generally less prevalent. Gentamicin resistance ranged from 0.0% in one study [61] to 10.0% in another [66] and 30.0% in Moroccan nasal isolates [59]. Gentamicin resistance was 61.4% in a study in Nigeria [60]. Tobramycin resistance was notably high in Moroccan isolates, with 60.0% of nasal isolates and 70.0% of hand isolates demonstrating resistance [59].

MDR prevalence varied across geographical regions. An Ethiopian study reported 53.0% of *Staphylococcus* spp. isolates exhibiting MDR [56]. A Nigerian research identified 35.7% of isolates as MDR, with 60.7% having a Multiple Antibiotic Resistance Index (MARI) greater than 0.2 [65]. In Malaysia, only 2 of 95 samples (2.1%) demonstrated MDR [58], while an Italian research found that 18.0% of isolates from anterior nares were multidrug resistant [44].

Egyptian food handler isolates showed extensive resistance to multiple antimicrobial classes, with resistance to 4–10 different antibiotics [36]. The most common resistance pattern observed in Nigerian isolates was simultaneous resistance to cefoxitin, oxacillin, and ciprofloxacin, occurring in 18.8% of isolates [65].

The genetic basis of antimicrobial resistance in *Staphylococcus* spp. isolates from food handlers revealed diverse resistance mechanisms. The *mecA* gene, encoding penicillin-binding protein 2a that confers methicillin resistance, was detected in varying frequencies: 18.9% of *S. aureus* isolates in Brazil [34], 100.0% of isolates from Egyptian food handlers [36], 25.5% from a Morrocco study [59], 7.4% from a Chinese study [63], and 5.0% from a Hong Kong study [66].

Beta lactamase production, mediated by the *blaZ* gene, was identified in 79.0% of Italian isolates resistant to penicillin G [44]. Additional resistance genes included *msrA* associated with erythromycin resistance, *linA* linked to clindamycin resistance, and *fusB* conferring resistance to fusidic acid in Italian isolates [44].

Beyond specific resistance genes, alternative resistance mechanisms were observed. An Egyptian research evaluated biofilm formation capability, identifying strong biofilm production in two isolates and moderate in six [36]. Biofilm formation enhances bacterial survival and increases resistance to antimicrobial agents and host immune defenses, which may help explain some resistance patterns not directly linked to specific resistance genes [67,68].

Antimicrobial resistance patterns also exhibited sample specific variations. In Morocco, nasal isolates showed higher resistance rates to gentamicin (30.0%), ciprofloxacin (50.0%), and cefoxitin (50.0%) compared to hands isolates, which demonstrated greater susceptibility to these antibiotics [59]. In another study, MRSA strains from nasal samples showed higher susceptibility to oxacillin and cefoxitin than those from hand samples [62].

The comprehensive analysis of *Staphylococcus* spp. colonization and antimicrobial resistance among food handlers reveals concerning patterns with meaningful public health implications. The significant geographical variation in MRSA prevalence, ranging from absent in some regions to ubiquitous in others, likely reflects a combination of factors, including differences in selective pressures, patterns of antimicrobial use, healthcare infrastructure, surveillance intensity, and population behaviors. Similarly, the considerable variation in resistance to critical antimicrobials, including vancomycin and linezolid, underscores the need for region-specific surveillance and targeted interventions.

The diversity of resistance mechanisms identified, including both genetic determinants and phenotypic adaptations like biofilm formation, demonstrates the remarkable adaptability of *Staphylococcus* spp. in food handling environments. The documented differences in colonization and resistance patterns between nasal and hand samples emphasize the importance of comprehensive sampling approaches when assessing colonization status and developing targeted decolonization strategies.

### 3.5. Outbreaks Linked to Antimicrobial Resistant S. aureus Isolated from a Food Handler

A 2019 *S. aureus* outbreak in an Italian nursing home was traced to chicken salad contaminated by an asymptomatic food handler. Laboratory analysis identified *S. aureus* ST72 isolates from the food handler’s nasal swab, chicken salad, and a patient’s vomit sample. All isolates carried *blaZ* and *norA* genes, with one strain from a second handler also harboring *ermC* [69].

A 2002 U.S. outbreak of methicillin-resistant *S. aureus* gastroenteritis was traced to coleslaw and barbecued pork prepared by an asymptomatic food handler. Pulsed field gel electrophoresis confirmed identical MRSA strains carrying the *mecA* gene in the handler’s nasal swab, coleslaw, and patients’ stool samples. The MRSA isolate was resistant only to penicillin and oxacillin [70].

### 3.6. Prevention and Control Strategies

*Staphylococcus* spp. exhibits remarkable environmental resilience, surviving on stainless steel, plastic, and fabric for days to weeks. In addition, biofilm formation enhances its persistence on food contact surfaces by resisting routine disinfection efforts [67,71]. *Staphylococcus* spp. biofilms on food contact surfaces play a critical role in antimicrobial resistance dissemination, as research has demonstrated that biofilm formation creates favorable conditions for the horizontal transfer of antimicrobial and biocide resistance genes to a variety of bacteria, both pathogenic and commensal, that may be present on food products or environmental surfaces within food production facilities [72].

Effective mitigation requires a multifaceted approach. Hand hygiene remains paramount: alcohol-based sanitizers reduce vegetative cells but must be paired with soap and water washing to ensure thorough removal of bacteria [73]. Given that fresh produce can harbor *Staphylococcus* spp. through environmental contamination or handler contact, rigorous washing of fruits and vegetables is crucial [74]. If gloves are used, they must be changed at certain intervals, such as while moving on to other work and after touching raw fruits and vegetables, and food handlers with wounds have to use gloves to prevent contamination [75,76]. Decolonization strategies, such as nasal and skin decolonization using mupirocin or chlorhexidine, can reduce *S. aureus* carriage. However, rising resistance to these agents necessitates novel approaches, including probiotics and targeting intestinal colonization [77,78].

Beyond hygiene protocols and decolonization strategies, targeted interventions such as food safety training and regular health screening play a critical role in preventing *S. aureus* transmission by food handlers [73,79]. Routine microbiological monitoring of hands can help assess the effectiveness of handwashing techniques, while nasopharyngeal and oropharyngeal screening is important for identifying asymptomatic *S. aureus* carriers [79,80]. Infected or colonized individuals should be temporarily excluded from food handling duties until treatment is completed, reducing the likelihood of contamination and foodborne outbreaks [73].

## 4. *Escherichia coli*

### 4.1. Overview of Escherichia coli

*Escherichia coli* is a Gram-negative, facultative anaerobic bacterium belonging to the *Enterobacteriaceae* family, ubiquitously found in the gastrointestinal tracts of humans and animals [81]. While most strains are harmless commensals, specific pathotypes have evolved virulence mechanisms enabling severe diseases [82]. Diarrheagenic *E. coli* strains are classified into six main categories based on intestinal pathogenicity: enterotoxigenic (ETEC), enteropathogenic (EPEC), enterohemorrhagic (EHEC), enteroaggregative (EAEC), enteroinvasive (EIEC), and diffusely adherent (DAEC). EHEC, particularly serotype O157:H7, is notorious for producing Shiga toxins (Stx1 and Stx2) [83]. Other virulence factors include intimin (a protein facilitating intestinal adherence) and heat stable and heat labile enterotoxins [84]. The plasticity of *E. coli* genomes, driven by horizontal gene transfer and acquisition of mobile genetic elements such as plasmids and transposons, facilitates rapid acquisition of antimicrobial resistance genes and virulence determinants, making it a formidable public health threat [85].

### 4.2. E. coli as a Common Foodborne Pathogen

Diarrheagenic *E. coli* strains are one of the leading causes of foodborne illnesses globally [86]. EHEC infections, often linked to undercooked beef or contaminated produce, manifest as abdominal cramps, bloody diarrhea, and, in some cases, hemolytic uremic syndrome, a life-threatening condition characterized by renal failure and thrombocytopenia [87]. ETEC, normally described as the biggest factor responsible for traveler’s diarrhea, produces heat labile and heat stable enterotoxins that induce watery diarrhea [88]. EPEC, prevalent in low resource settings, adheres to intestinal epithelia via bundle forming pili, causing effacement of microvilli and persistent diarrhea in children [89]. EAEC colonizes intestinal mucosa, secreting cytotoxins that provoke inflammatory responses, while EIEC invades colonic epithelial cells, mimicking dysentery caused by *Shigella* [90,91]. DAEC has been implicated in persistent diarrhea in pediatric populations, particularly in low resource settings [92].

### 4.3. Role of Food Handlers in E. coli Colonization and Transmission

*E. coli* colonization begins in the human gut shortly after birth, with commensal strains playing roles in nutrient metabolism and pathogen exclusion [93]. Pathogenic strains, however, employ adhesins like intimin (in EHEC and EPEC) or aggregative adherence fimbriae (in EAEC) to bind host receptors, evading peristalsis [91,94]. Biofilm formation on abiotic surfaces, such as stainless steel, plastic, and food processing equipment, enhances environmental persistence [95]. Studies show that *E. coli* can survive for weeks on dry surfaces and resist disinfectants, such as quaternary ammonium compounds, when embedded in biofilms [96]. In food production environments, inadequate sanitation allows biofilm dispersal, contaminating products like leafy greens, dairy, and meats [97,98]. The bacterium’s ability to enter a viable but non culturable (VBNC) state under stress further complicates detection and eradication [99].

*E. coli*’s ubiquity in animal reservoir and its fecal oral transmission route make it a common foodborne pathogen [100]. *E. coli* contamination of food products can occur through multiple pathways, including fecal contamination during slaughter processes, where intestinal contents contact meat surfaces; use of contaminated water for crop irrigation; cross-contamination in food preparation environments via improperly sanitized utensils or surfaces; and improper hand hygiene among food handlers, enabling direct transfer of pathogenic strains to ready-to-eat foods [101]. High risk foods include undercooked ground beef, raw milk, unpasteurized juices, and fresh produce [102].

Food handlers serve as critical sources for *E. coli* transmission when basic hygiene protocols are neglected [103]. Contaminated hands can transfer pathogenic strains to ready-to-eat foods through direct contact, particularly when handlers fail to wash hands after using the restroom, handling raw ingredients, or touching contaminated surfaces [104]. The fecal oral pathway is particularly important, as *E. coli* shed in feces can persist on hands and surfaces, enabling transfer to food during preparation [105]. In a study of 173 food handlers in Indonesian campus canteens, improper handwashing techniques and lack of knowledge about food as a disease medium were strongly correlated with *E. coli* contamination in served meals. Specifically, food handlers who practiced high risk handwashing behaviors (infrequent soap use) had 0.082 times lower odds of contamination, indicating that lapses in hand hygiene directly facilitated bacterial transfer to food [106].

In street vending environments, where access to clean water is limited, *E. coli* contamination is more likely, and cross-contamination between hands and various utensils is exacerbated [107]. Shared equipment, such as knives, cutting boards, or packaging materials, facilitates indirect transmission if not properly sanitized between uses [108]. Storage practices also contribute meaningfully [56]. A Malaysian study revealed that cooks, who routinely handle raw meat and vegetables, had higher stool contamination levels than waiters, suggesting that their frequent exposure to pathogens increases the risk of transmitting infections through cross-contaminated tools or surfaces [109]. This underscores the importance of rigorous hand hygiene practices among food handlers to prevent pathogen dissemination [73].

Asymptomatic food handlers colonized with *E. coli* pose a stealth transmission risk, as they shed pathogens without exhibiting symptoms [110]. A German case further demonstrated this: a catering worker unknowingly infected with STEC O104:H4 prepared food for 71 guests, resulting in 23 laboratory confirmed cases. Despite reporting no symptoms at the time of food preparation, subsequent stool testing revealed persistent colonization, underscoring the danger of asymptomatic shedding in communal settings [111].

Food handlers often mediate zoonotic transmission through contact with raw, animal-derived foods. *E. coli*, which is naturally present in the digestive systems of most animals sent for slaughter, contaminates meat during processing [112]. Handlers who touch raw beef or can transfer *E. coli* to ready-to-eat foods [113]. Close-proximity food preparation environments enable person-to-person transmission among handlers. *E. coli* spread through shared workspaces when contaminated hands touch common surfaces [114,115]. Airborne transmission may also disseminate pathogens to uncovered foods and foods [116].

### 4.4. Antimicrobial Resistance in E. coli Isolated from Food Handlers

Table 2 represents the major results from most of the studies utilized in this review on antimicrobial resistance in *E. coli* isolated from food handlers worldwide.

The global prevalence of *E. coli* colonization among food handlers exhibits extreme variability, with studies reporting rates from 2.8% in Malaysia [61] to 89.3% in China [120]. The Chinese study identified 92 isolates from 103 handlers, representing the highest documented colonization rate in this review, likely reflecting regional differences in hygiene infrastructure or environmental contamination [120]. At the lower end, Malaysian and Kenyan studies reported rates of 2.8% (28/1020) and 4.4% (39/885), respectively [61,122]. High burden regions included Kuwait, where 62.4% (425/681) of bacterial isolates from 405 handlers were *E. coli* [15], and Tunisia, which identified 378 ESBL producing strains from 2135 samples [123].

Investigations that included healthcare settings as sampling sites revealed substantially higher *E. coli* prevalence among food handlers (41.4–89.3%) compared to studies conducted exclusively in non-healthcare facilities (2.8–54.1%). The higher levels of antimicrobial resistance observed in healthcare environments compared to other settings imply that hospitals constitute unique ecological niches with enhanced antimicrobial exposure [124]. Such heightened selective pressure may alter microbial carriage patterns among food handlers, potentially increasing their risk of colonization with resistant strains.

Antimicrobial susceptibility profiles revealed stark contrasts, particularly for β-lactams. Ampicillin resistance ranged from 32.1% in Qatar [14] to total resistance (100.0%) in Moroccan isolates [121]. Ciprofloxacin resistance spanned from 7.7% in Kenya [122] to 75.0% among Gambian ESBL producers [118], while nalidixic acid resistance peaked at 88.9% in Morocco [121] and 65.2% in China [120]. Tetracycline resistance dominated across regions, affecting 75.7% of Tunisian ESBL isolates [123] versus 53.8% in Kenya [122], 67.4% in China [120], with cotrimoxazole resistance exceeding 50.0% in Gambia and Tunisia [118,123].

ESBL production rates varied, with Kuwait reporting 18.8% (80/425) of isolates as ESBL producers [15], Ethiopian studies ranging from 17.6% [115] to 24.4% [117] and Tunisia showing 17.7% ESBL prevalence [123]. A study from Gambia identified carbapenemase production in 12.5% (1/8) of ESBL isolates [118]. In the same vein, an Ethiopian study reported four *E. coli* strains producing carbapenemase, highlighting a concerning emergence of carbapenem resistance [115]. Morocco reported an alternative resistance mechanism, with 38.9% (7/18) of isolates producing metallo-β-lactamases (MBLs) despite lacking ESBL genes, highlighting diverse β-lactam resistance pathways [121].

Genetic characterization uncovered region-specific resistance determinants. Tunisian ESBL producers predominantly carried *bla*_CTX-M-15_ (57.9%), followed by *bla*_CTX-M-1_ (18.5%) and *bla*_CTX-M-27_ (13.8%) [123], while Chinese isolates harbored *bla*_CTX-M-14_ (71.4% of ESBL strains) [120]. Plasmid mediated quinolone resistance genes (*qepA1, qnrS1, qnrB6*) emerged in 4.3% of Chinese isolates, complementing chromosomal mutations driving high nalidixic acid resistance (65.2%) [120].

MDR prevalence ranged from 14.3% in Malaysia [61] to 88.9% in Morocco [121] and 100.0% in Gambia [118], with critical resistance clusters identified globally. The Gambian ESBL isolates demonstrated resistance to ceftriaxone, cefotaxime, ampicillin, and tetracycline (100.0%), coupled with 75.0% ciprofloxacin resistance [118]. Indonesia reported a high proportional MDR burden (83.3%, 20/24), while Kuwait documented 30.6% (130/425) MDR prevalence, with MIC90 values exceeding 256 μg/mL for ampicillin and tetracycline [15,119].

Mobile genetic elements facilitated resistance dissemination, particularly in high prevalence regions. Class 1 integrons (intI1) were detected in 50.0% of Chinese isolates, promoting horizontal gene transfer [120]. Tunisia identified the global high-risk clone ST131 in 13.2% (50/378) of ESBL producers, indicating clonal expansion within food handling populations [123].

Risk factor analysis in a study in Ghana revealed that untrained handlers had twice the likelihood of carrying resistant bacteria, while open defecation practices increased risk sixfold. Paradoxically, recent antibiotic usage reduced carriage odds, potentially due to suppression of susceptible flora favoring resistant strain persistence. These findings underscore the complex interplay between hygiene infrastructure, antimicrobial exposure, and resistance gene flow in food handling environments [125].

The resilience of *E. coli* stems from its genetic adaptability and environmental hardiness. Horizontal gene transfer facilitates the spread of resistance elements like ESBL and *mcr-1* across bacterial species [126]. Biofilms in food processing plants resist chlorine-based sanitizers, necessitating advanced disinfection methods (e.g., phage therapy, cold plasma) [95]. The global food trade serves as an important actor for the dissemination of antimicrobial resistant bacteria, as evidenced by genomic analyses revealing striking similarities between resistance plasmids found in food products and those previously identified in diverse geographical regions [127].

### 4.5. Prevention and Control Strategies

Effective management of antimicrobial-resistant *E. coli* transmission via food handlers necessitates a multifaceted approach integrating personal hygiene, environmental controls, and systemic interventions. Hand hygiene remains the cornerstone of prevention [128], as does implementation of the World Health Organization’s “Five Keys to Safer Food” protocol, emphasizing clean hands, separation of raw and cooked foods, thorough cooking, safe temperatures, and clean water/raw materials [129].

Environmental interventions must address the persistence of resistant *E. coli* in food preparation settings [130]. Surface sanitization using quaternary ammonium compounds or hydrogen peroxide-based disinfectants effectively reduces bacterial loads on high touch surfaces, though biofilm embedded bacteria may require more aggressive approaches [131,132]. Time-temperature control during food preparation and storage presents another critical intervention point that must be adhered to properly by food handlers [133]. Comprehensive training programs for food handlers that emphasize the invisible nature of microbial contamination and the specific risks of antimicrobial resistant pathogens have demonstrated sustained improvements in compliance with hygiene protocols [107,134]. The integration of these personal, environmental, and educational strategies creates a robust defense against the transmission of antimicrobial resistant *E. coli* in food service environments, protecting both consumers and the broader community from these increasingly prevalent pathogens [135,136,137].

## 5. *Salmonella* spp.

### 5.1. Overview of Salmonella *spp.*

*Salmonella* spp. is a Gram-negative, facultative anaerobic bacterium within the *Enterobacteriaceae* family, comprising two primary species: *Salmonella enterica* and *Salmonella bongori*. *S. enterica*, the most clinically relevant species, is further classified into over 2600 serovars, with *Salmonella* Typhimurium and *Salmonella* Enteritidis being the most frequently implicated in human infections [138,139]. These pathogens are characterized by their flagellar motility, ability to invade host cells, and robust environmental persistence [139,140]. *Salmonella* spp. employs virulence mechanisms encoded by pathogenicity islands (SPI-1 and SPI-2), which facilitate epithelial cell invasion and intracellular survival [141]. SPI-1 associated Type III Secretion Systems (T3SS) inject effector proteins into host cells, disrupting cytoskeletal structures to promote bacterial uptake, while SPI-2 supports replication within phagosomes by modulating host immune responses [142]. Non typhoidal *Salmonella* (NTS) serovars typically cause self-limiting gastroenteritis, but invasive strains can lead to systemic infections, particularly in immunocompromised individuals [143]. In contrast, typhoidal serovars like *Salmonella* Typhi and *Salmonella* Paratyphi are adapted to human hosts, causing typhoid fever, a life-threatening systemic illness endemic in regions with poor sanitation and limited access to clean water [144].

### 5.2. Salmonella *spp.* as a Common Foodborne Pathogen

Non-typhoidal *Salmonella* infections are one of the leading causes of foodborne illness globally, manifesting as acute gastroenteritis with symptoms including diarrhea, abdominal cramps, fever, and vomiting, typically occurring 12 to 96 h after ingestion of contaminated food [145,146]. Invasive NTS strains, particularly prevalent in sub–Saharan Africa among populations with high HIV prevalence or malnutrition rates, can lead to bacteremia, meningitis, and osteomyelitis [147]. Typhoidal *Salmonella*, transmitted via the fecal oral route, causes prolonged fever, headache, and gastrointestinal disturbances, with more than 25 million cases and more than 200,000 deaths annually worldwide [148,149,150]. The global burden of salmonellosis underscores its public health importance, with NTS alone responsible for an estimated 93.8 million illnesses with 85.6% being foodborne [151].

### 5.3. Role of Food Handlers in Salmonella *spp.* Colonization and Transmission

*Salmonella* spp. colonization begins with oral ingestion of contaminated food or water [152]. The bacteria survive gastric acidity through acid tolerance response mechanisms and adhere to intestinal epithelial cells via fimbriae and adhesins [153,154]. SPI-1 mediated T3SS facilitates invasion into epithelial cells, suppressing early proinflammatory cytokine expression in macrophages and helps replication within host cells [155]. *Salmonella* spp. achieves systemic dissemination through several key routes: by invading M cells in Peyer’s patches, by being transported within dendritic cells and phagocytes to lymph nodes and bloodstream, and by establishing persistent infection in macrophages and epithelial cells that can serve as reservoirs for later dissemination to organs such as the liver, spleen, and gallbladder [152]. Environmental resilience is a hallmark of *Salmonella* spp., which persists for weeks on surfaces like stainless steel, plastic, and glass, particularly in moist conditions [156,157,158]. Like in other bacteria previously mentioned in this article, *Salmonella* spp. biofilm formation enhances resistance to disinfectants, while the ability to enter a VBNC state under stress complicates detection in food processing environments [159].

The ubiquity of *Salmonella* in animal reservoirs, including poultry, cattle, and swine, facilitates its entry into the food supply chain [160]. Contamination often occurs during slaughter via fecal contact, cross-contamination in processing facilities, or through irrigation water as well as recycled water used in machinery and manure [161,162]. High risk foods include undercooked poultry and pork, soft boiled eggs, raw milk, and fresh produce such as vegetables and fruits [138,163,164].

Food handlers serve as critical contamination sources of *Salmonella* spp., primarily through suboptimal hygiene practices [138]. Asymptomatic carriers exacerbate this risk, particularly when handwashing protocols are inadequately followed, leading to contamination of ready-to-eat foods [165]. Indirect transmission routes further amplify dissemination, as pathogens spread via contaminated surfaces, utensils, or food items exposed to improperly handled materials during preparation [166]. Socioeconomic determinants, including disparities in infrastructure and regulatory enforcement, influence regional infection rates [167]. Populations such as street vendors and institutional food handlers face heightened vulnerability due to overcrowded workspaces and inconsistent adherence to safety protocols [168].

Empirical evidence highlights the critical role of personal hygiene in pathogen carriage. In Ethiopian cohorts, food handlers who neglected handwashing with soap after defecation had 3.3 times higher odds of *Salmonella* spp. infection, while those with untrimmed fingernails faced a 4.4 times increased risk [165]. A complementary study in Ethiopia revealed a marked disparity: pathogens infections were 1.84 times more likely to occur among food handlers who had untrimmed fingernail as compared to those who trimmed [169]. Although glove usage can mitigate transmission, improper practices, such as failing to change gloves between raw and ready-to-eat food handling, paradoxically promote cross-contamination [170].

Behavioral factors further modulate risk. Educational interventions improve compliance with hygiene standards, yet an optimism bias among food handlers may undermine precautions [171]. This cognitive tendency, wherein individuals perceive themselves as less susceptible than others to causing foodborne illness, correlates with training participation but may reduce vigilance. Overly optimistic handlers are more likely to neglect protective measures, thereby increasing contamination risks [172].

### 5.4. Antimicrobial Resistance in Salmonella *spp.* Isolated from Food Handlers

Emerging evidence highlights food handlers as contamination sources of antimicrobial resistant *Salmonella* spp., with substantial variations in contamination rates and resistance profiles across geographical regions. The studies that analyzed *Salmonella* spp. antimicrobial resistance from food handlers are described on Table 3.

Surveillance data demonstrate pronounced disparities in *Salmonella* spp. prevalence among food handlers, ranging from 0.07% in a study in Japan (2012–2022) [184] to 9.1% in Pakistan [183]. Asian nations exhibit divergent trends: large scale screening in China (214,542 samples) identified 0.09% positivity [13], while Malaysia reported 2.8% prevalence among 317 workers [109]. Japan’s decade long monitoring of 27.8 million individuals revealed exceptionally low contamination (0.07%), contrasting sharply with Ethiopia’s 5.13% prevalence across 3140 food handlers [56,165,173,174,175,176,177,178,179,180]. These disparities likely reflect differences in hygiene protocols, antimicrobial stewardship, and diagnostic sensitivity between regions.

The prevalence and serovar distribution of *Salmonella* among food handlers varied notably across studies, reflecting regional epidemiological patterns. In Japan, Sasaki et al. [181] isolates were dominated by *S*. Schwarzengrund (17.0%) and *S*. Infantis (8.6%), with chicken-derived isolates showing a striking predominance of *S*. Schwarzengrund (73.0%), suggesting poultry as a key reservoir. Similarly, Shigemura et al. [182] reported *S*. Infantis (12.7%) and *S*. Schwarzengrund (9.5%) as prevalent serotypes among 158 isolates, reinforcing the persistence of these serotypes in Japanese food systems. Another study in Japan, showed a shifting serotype dominance, *S*. enteritidis was predominant in 2006, *S*. Infantis in 2012, *S*. Agoueve/Cubana in 2018, and *S*. Schwarzengrund in 2021 [184]. In contrast, Siddiqui et al. [183] in Pakistan documented a high proportion of typhoidal serovars (47.4%), including *S*. Typhi (36.8%) and *S*. Paratyphi A/B (10.6%), highlighting endemic typhoid transmission linked to human carriers. Xu et al. [13] in China observed a distinct profile among 193 isolates, with *S*. Typhimurium (16.1%) and *S*. Derby (13.5%) predominating, likely reflecting zoonotic transmission from pork, a staple meat in the country. Woh et al. [109] in Malaysia isolated nine strains, primarily *S*. Typhimurium (33.3%) and *S*. Corvallis (22.2%).

Longitudinal Japanese data (2012–2022) uncovered annual increases in resistance to cefotaxime, ceftazidime, nalidixic acid, kanamycin, streptomycin, and tetracycline. Streptomycin resistance remained persistently high (32.0–39.0%), while tetracycline resistance escalated from 17.0% to 37.0% over the study period. These trends underscore the urgency of continuous antimicrobial resistance surveillance in food safety systems [184].

Ampicillin resistance dominated in China (64.6%) [13] and Pakistan (77.7–100%) [183], whereas Japanese isolates exhibited elevated streptomycin (32.0–51.1%) and tetracycline (17.0–39.2%) resistance [181,182,184]. China reported substantial sulfisoxazole (58.1%) and nalidixic acid (55.8%) resistance, suggesting regional prescribing practices influence resistance selection [13].

MDR prevalence varied markedly: Ethiopia reported 54.7% MDR among isolates [56,165,173,174,175,176,177,178,179,180], followed by China (73.4%) [13] and Malaysia (77.8%) [109]. Japan showed moderate MDR rates (40.3%) [181], while Pakistan documented only one MDR isolate [183]. These disparities may reflect differential antibiotic regulation stringency, with lower income nations demonstrating higher MDR burdens.

Japanese surveillance identified ESBL production in 0.8–5.3% and AmpC β-lactamase in 1.8–4.1% of isolates [182,184]. The *bla*_CMY-2_ gene predominated in AmpC producers, while ESBL strains carried *bla*_CTX-M-14_, bla_CTX-M-15_, *bla*_SHV_, and *bla*_TEM_.

A landmark Chinese study identified the first human *Salmonella* 4,[5],12:i:- isolate carrying *mcr-1*, *bla*_CTX-M-14_, and *fosA3* on a conjugative IncHI2 plasmid (pYZU1189). This finding confirms horizontal gene transfer potential between food handlers and consumers, exacerbating resistance dissemination risks [185].

The collective evidence from studies across multiple countries reveals substantial geographic variation in both the prevalence of *Salmonella* spp. among food handlers and the antimicrobial resistance profiles of these isolates. The highest resistance rates were observed for ampicillin, tetracycline, and streptomycin, though the specific resistance patterns differed by region. MDR *Salmonella* spp. was particularly prevalent in Ethiopia, Malaysia, and China, while genetic analysis revealed several β-lactamase genes, with *bla_CMY-2_* and *bla_CTX-M_* variants being the most commonly identified. The alarming rates of MDR *Salmonella* spp. carriage among food handlers across different regions highlight the potential role of food service workers in the dissemination of antimicrobial resistant pathogens. These findings underscore the need for enhanced surveillance, improved hygiene practices, and prudent antimicrobial use policies to mitigate the spread of resistant *Salmonella* spp. through the food chain. The identification of transferable resistance plasmids, as reported in the Chinese study [185], further emphasizes the public health relevance of this issue, as these mobile genetic elements can facilitate the rapid spread of resistance determinants across bacterial populations.

### 5.5. Outbreak Linked to Antimicrobial Resistant Salmonella *spp.* Isolated from a Food Handler

In 2023, a *S.* Enteritidis outbreak occurred at a Chinese restaurant, affecting 26 laboratory-confirmed cases among 31 exposed customers. The epidemiological investigation established genetic identity among isolates obtained from patients (n = 2), contaminated partially processed food components (n = 2), and one asymptomatic food service worker (n = 1). Antimicrobial testing showed consistent resistance patterns across isolates to nalidixic acid (100.0%) and colistin (80.0%), while reduced susceptibility to ciprofloxacin was linked to the *gyrA* p.D87G mutation that confers decreased fluoroquinolone sensitivity. Genetic analysis revealed 107 virulence factors, including the crucial SPI-1 and SPI-2 pathogenicity islands essential for bacterial invasion and survival within host cells. The transmission investigation definitively identified an asymptomatic food handler as the outbreak source, highlighting deficiencies in existing food safety measures and employee health surveillance programs [186].

### 5.6. Prevention and Control Strategies

Mitigating *Salmonella* spp. contamination requires a multifaceted approach. Farm level interventions include probiotics and bacteriophage therapies to reduce colonization in poultry [187,188]. Vaccination programs employing live attenuated *Salmonella* spp. strains and bacterins have considerably reduced *Salmonella* spp. transmission into eggs by inducing robust mucosal and systemic immunity in layer hens, thereby decreasing egg contamination and vertical transmission of *Salmonella* spp. [189]. Effective treatments to disrupt *Salmonella* spp. biofilms in food processing plants include natural antimicrobials (plant extracts, essential oils), enzymatic approaches (proteinase K, flavourzyme, mixed enzyme complexes), biological controls (lactic acid bacteria, bacteriocins, bacteriophages), and physical methods (ionizing radiation, cold plasma, ultrasound), with combined treatments showing synergistic effects superior to individual applications [190]. Consumer education initiatives focusing on the four safe food handling practices of “clean, separate, cook, and chill” have demonstrated effectiveness in reducing cross-contamination risks [191].

The genetic plasticity of *Salmonella* spp. presents major challenges for controlling resistant strains, as the bacterium readily acquires resistance through horizontal gene transfer via plasmids, while its ability to form biofilms on food processing surfaces enhances environmental persistence and reduces antibiotic penetration [192]. The global food trade further complicates antimicrobial resistance control, as resistant *Salmonella* strains can spread across international borders through contaminated food products, with studies showing plasmid mediated resistance genes such as *mcr-1* (colistin resistance) being detected in both retail poultry and human clinical isolates, underscoring the food chain’s role as a reservoir for resistance dissemination [193].

Regular handwashing with soap and water, particularly after restroom use and handling raw ingredients, reduces contamination risk, while maintaining short, clean fingernails decreases pathogen carriage likelihood [194,195]. Proper glove usage, changing between tasks and avoiding cross-contamination between raw and ready-to-eat foods is critical [195,196]. Routine health screenings to identify asymptomatic carriers, coupled with mandatory exclusion policies for infected workers during shedding periods, are essential to break transmission chains [109,171]. Behavioral interventions, including culturally adapted training and the risks of antimicrobial resistant strains can improve food safety [175,197]. Regulatory enforcement of food safety certifications, coupled with microbial surveillance of hands and food contact surfaces in high-risk settings (e.g., street vending), may reduce multidrug resistant *Salmonella* prevalence [198]. These measures must be integrated with existing farm-to-fork strategies to address the dual threat of pathogen dissemination and antimicrobial resistance amplification [199].

## 6. Comparative Analysis of Food Handler Associated Pathogens

The examination of colonization dynamics across *Staphylococcus* spp., *E. coli*, and *Salmonella* spp. reveals distinct epidemiological patterns with critical public health implications. Geographical distribution analysis demonstrates that *Staphylococcus* exhibits the widest colonization range among food handlers, with prevalence rates spanning from 19.5% in Myanmar to 95.0% in Malaysia [35,58]. This contrasts sharply with *Salmonella*’s more restricted prevalence spectrum, ranging from 0.07% in Japan to 9.1% in Pakistan [183,184]. *E. coli* displays intermediate variability, with Chinese studies reporting hand contamination rates as high as 89.3%, while Malaysian investigations document much lower rates of 2.8% [61,120]. These disparities likely reflect differences in anatomical colonization niches, with *Staphylococcus* spp. inhabiting nasal mucosa and hands, *E. coli* maintaining fecal oral transmission cycles, and *Salmonella* spp. showing intestinal persistence through fecal shedding.

Antimicrobial resistance profiles exhibit pathogen specific trends that mirror their genetic adaptability. *Staphylococcus* spp. demonstrates near universal β-lactam resistance (57.9–100.0% penicillin resistance across studies) [58,104], while maintaining variable susceptibility to last resort agents like vancomycin (0.0–72.9% resistance) [34,62]. *E. coli* shows concerning plasmid mediated resistance mechanisms, with ESBL production rates ranging from 17.6% in Tunisia [123] to 24.4% in one study in Ethiopia [117], and the emergence of carbapenem resistance, reported at 1.6% in an Ethiopian study [115] and 12.5% among ESBL isolates from Gambia [118]. *Salmonella* spp. displays particularly high ampicillin resistance (64.6–100.0%) [13,183], coupled with important streptomycin resistance in Japanese studies (32.0–51.1%) [181,182,184]. The convergence of MDR across all three pathogens presents a sobering reality: while Malaysian *Staphylococcus* spp. isolates show only 2.1% MDR prevalence [58], Malaysian *Salmonella* strains reach 77.8% MDR rates [109], and Moroccan *E. coli* isolates demonstrate 88.9% multidrug resistance [121].

Transmission dynamics diverge according to pathogen ecology and handler behavior. *Staphylococcus* spp. transmission primarily occurs through nasal droplet dispersion and surface contamination, facilitated by biofilm formation on kitchen implements [47,48,51]. *E. coli* dissemination mainly follows classic fecal oral pathways, exacerbated by inadequate handwashing after restroom use [104,105]. *Salmonella* spp. transmission combines zoonotic and environmental routes, with Japanese surveillance data revealing food handler contamination linked to poultry handling practices [13,181,192]. All three pathogens exploit food handler asymptomatic carriage, though with differing persistence mechanisms.

## 7. Way Forward

Addressing the global challenge of antimicrobial-resistant foodborne pathogens necessitates innovative, multidisciplinary strategies anchored in robust surveillance and targeted interventions. Future efforts should prioritize the integration of genomic sequencing and real time data sharing to track resistance patterns and emerging strains across regions, enabling proactive responses. Enhanced disinfection technologies, such as phage therapy and cold plasma, should be developed to combat biofilm persistence on food contact surfaces, while standardized hygiene protocols must be universally adopted in food service environments. Investment in culturally adapted training programs for food handlers, emphasizing hand hygiene and cross-contamination prevention, is critical to reducing pathogen transmission. Policymakers must enforce stricter regulations on antibiotic use in agriculture and food production to curb resistance selection. Additionally, One Health frameworks should be expanded to bridge gaps between human health, veterinary, and environmental sectors, fostering collaboration to disrupt resistance gene flow. By harmonizing technological advances, education, and policy reform, the global community can mitigate the escalating threat posed by resistant pathogens in the food chain.

Although this review aimed to provide a global assessment of antimicrobial resistant *Staphylococcus* spp., *E. coli*, and *Salmonella* spp. in food handlers, the majority of studies identified were conducted in low- and middle-income countries. No geographical restrictions were applied during the literature search; however, there is a notable lack of published studies from high-income countries. It is important to recognize that the scarcity of data from regions with more stringent hygiene standards and food safety regulations may limit the generalizability of some findings. Future research efforts should address this gap to better inform globally representative risk assessments and policy development.

## 8. Conclusions

The persistence and transmission of antimicrobial resistant *Staphylococcus* spp., *Escherichia coli*, and *Salmonella* spp. through food handlers underscore a critical nexus between human behavior, microbial adaptability, and global food safety systems. These pathogens exploit gaps in hygiene infrastructure, asymptomatic colonization, and environmental resilience to propagate resistance traits, with biofilm formation, horizontal gene transfer, and stress induced survival mechanisms further complicating eradication efforts.

Food handlers, as frontline actors in food production chains, are uniquely positioned to either mitigate or amplify the spread of these resistant pathogens. Their frequent contact with raw ingredients, shared surfaces, and ready-to-eat foods creates opportunities for cross-contamination, particularly when hygiene protocols are inconsistently practiced. Asymptomatic carriers further enable silent transmission, while socioeconomic disparities in training and resource access exacerbate risks in low- and middle-income settings. Addressing these challenges requires prioritizing food handlers through targeted interventions. Strengthening antimicrobial stewardship in agriculture and healthcare, alongside policies that incentivize compliance with food safety standards, will reduce selective pressures driving resistance. Implementing active surveillance strategies, such as periodic screening of asymptomatic food handlers, is an interesting approach to prevent silent transmission of resistant pathogens. By recognizing food handlers as both critical safeguards and potential vulnerabilities in the AMR landscape, global efforts can disrupt transmission pathways, safeguarding food systems and public health against the escalating threat of untreatable infections.

## Figures and Tables

**Table 1:** Prevalence and antimicrobial resistance profiles of *Staphylococcus* spp. isolated from food handlers worldwide.

Country	Location of Collection	Isolates from Food Handler (%)	Sample	Antimicrobial Factors	Resistance Genes	MDR (%)	Reference
Ethiopia	Multiple	181/384 (47.1%)	Hand	Not analyzed	Not analyzed	96/181 (53.0%)	[56]
Iran	Not informed	224/1113 (20.1%)	Nasal	37 MRSA	Not analyzed	Not analyzed	[57]
Brazil	Pilot kitchen	74/82 (90.2%)	Underside of nails and nasal	7 MRSA	7 *mecA*	Not informed	[34]
Myanmar	Hotel and Restaurant	144/563 (25.6%)	Hands and Nasal	Not analyzed	Not analyzed	2/144 (1.4%)	[35]
Egypt	Not informed	12/40 (30.0%)	Hand	12 MRSA 3 VRSA	12 *mecA* 3 *vanA* 2 *vanB*	12/12 (100.0%)	[36]
Malaysia	Not informed	95/100 (95.0%)	Hand	Not analyzed	Not analyzed	2/95 (2.1%)	[58]
Italy	Pasta company	7/28 (25.0%)	Hand and nasal	Not analyzed	22 *blaZ* 9 *msrA* 1 *linA* 1 *fusB*	5/28 (18.0%)	[44]
Morrocco	Hospital	55/70 (78.6%)	Hand and nasal	Not analyzed	13 *mecA*	51/51 (100.0%)	[59]
Nigeria	Street food handlers	101/360 (28.1%)	Hand and nasal	22 MRSA	Not analyzed	96/101 (95.0%)	[60]
Malaysia	School	179/1020 (17.5%)	Hand	Not analyzed	Not analyzed	1/148 (0.7%)	[61]
Brazil	Hospital	111/280 (39.6%)	Hand and nasal	40 MRSA	Not analyzed	Not informed	[62]
China	Not informed	231/1927 (12.0%)	Hand	17 MRSA	17 *mecA*	17/231 (7.4%)	[63]
Lebanon	Not informed	38/160 (23.8%)	Nasal	5 MRSA	Not analyzed	Not informed	[64]
Nigeria	Roadside food handlers	28/180 (15.6%)	Hand and Nasal	Not analyzed	Not analyzed	10/28 (35.7%)	[65]
Hong Kong	Catering establishments	99/434 (22.8%)	Nasal	5 MRSA	5 *mecA*	Not informed	[66]

MDR, (multidrug resistant); MRSA (methicillin-resistant *S. aureus*); VRSA (vancomycin-resistant *S*. *aureus*).

**Table 2:** Prevalence and antimicrobial resistance profiles of *E. coli* isolated from food handlers worldwide.

Country	Location of Collection	Positive Isolates from Food Handler (%)	Sample Type	Antimicrobial Mechanisms	Resistance Genes	MDR (%)	Reference
Ethiopia	Multiple	95/384 (24.7%)	Hand and Fecal	Not analyzed	Not analyzed	56/95 (59.0%)	[56]
Ethiopia	University cafeterias (Including Hospital)	245/290 (84.5%)	Fecal	43 ESBL 4 Carbapenemase	Not analyzed	104/245 (42.4%)	[115]
Ethiopia	University cafeterias	119/220 (54.1%)	Fecal	29 ESBL	Not analyzed	27/119 (22.7%)	[117]
Qatar	Migrant food handlers during mandatory medical screening	78/456 (17.1%)	Fecal	7 ESBL	Not analyzed	21/78 (27.0%)	[14]
Kuwait	Commercial eateries and Healthcare settings	425/681 (62.4%)	Fecal	80 ESBL	Not analyzed	130/425 (30.6%)	[15]
Gambia	Schools	8 ESBL producing *E. coli*/565 *	Fecal	8 ESBL 4 AmpC 1 Carbapenemase	Not analyzed	8/8 (100.0%)	[118]
Indonesia	Hospitals	24/58 (41.4%)	Hand and Nasal	Not analyzed	Not analyzed	20/24 (83.3%)	[119]
Malaysia	Schools	28/1020 (2.8%)	Hands	Not analyzed	Not analyzed	4/28 (14.3%)	[61]
China	Military hospital	92/103 (89.3%)	Fecal	7 ESBL 46 intI1 2 qepA1 1 qnrS1 1 qnrB6	5 *bla_CTX-M14_* 1 *bla_CTX-M79_* 1 *bla_CTX-M-106_*	47/92 (51.1%)	[120]
Morroco	Hospital	18/40 (45.0%)	Hands	ESBL not detected 16 metallo-β-lactamase	Not analyzed	16/18 (88.9%)	[121]
Kenya	Hotels	39/885 (4.4%)	Fecal	Not analyzed	Not analyzed	16/39 (40.2%)	[122]
Tunisia	Not mentioned	378 ESBL producing *E. coli*/2135 *	Fecal	378 ESBL	219 *bla_CTX-M-15_* 70 *bla_CTX-M-1_* 52 *bla_CTX-M-27_* 23 *bla_CTX-M-14_* 10 *bla_SHV-12_* 3 *bla_SHV-2a_* 1 *bla_CTX-M-3_*	Not informed	[123]

MDR (multidrug resistant); ESBL (extended spectrum β-lactamase). * Quantity of *Escherichia coli* isolates was not provided; this study screened for ESBL and then confirmed the bacteria.

**Table 3:** Prevalence and antimicrobial resistance profiles of *Salmonella* spp. isolated from food handlers worldwide.

Country	Location of Collection	Isolates from Food Handler (%)	Sample	Antimicrobial Factors	Resistance Genes	MDR (%)	Reference
Ethiopia *	Multiple	161/3140 (5.1%)	Fecal and Hand	Not analyzed	Not analyzed	88/161 (54.7%)	[56,165,173,174,175,176,177,178,179,180]
China	Catering industry	193/214,542 (0.09%)	Fecal	Not analyzed	Not analyzed	85/116 (73.4%)	[13]
Japan	Cooks and servers in restaurants and food factory workers	583/740,635 (0.079%)	Fecal	Not analyzed	5 *bla*_CMY-2_ 2 *bla*_CTX-M-15_ 1 *bla*_LAT-3_ 3 *bla*_CTX-M-55_ 1 *bla*_TEM-52B_ 1 *bla*_LAP-2_ 1 *bla*_TEM-1_	110/273 (40.3%)	[181]
Japan	Cooks and servers in restaurants and food factory workers	164/145,220 (0.113%)	Fecal	4 ESBL and 3 AmpC-lactamase	1 *bla*_CTX-M-14_ 3 *bla*_CTX-M-15_ 3 *bla*_CMY-2_	Not informed	[182]
Malaysia	Food establishments	9/317 (2.8%)	Fecal	Not analyzed	Not analyzed	7/9 (77.8%)	[109]
Pakistan	Food street vendors	19/209 (9.1%)	Fecal	Not analyzed	Not analyzed	1/19 (5.3%)	[183]
Japan	Not mentioned	19 602/27,848,713 (0.07%)	Fecal	3/400 ** ESBL 7/400 ** AmpC	7 *bla*_CMY-2_ 1*bla*_TEM_ 1 *bla*_SHV_	Not informed	[184]

MDR (multidrug resistant); ESBL (extended spectrum β-lactamase). * Consolidated data from multiple Ethiopian studies, presented in a single row for clarity. ** Only a subset of the total *Salmonella* spp. positive isolates was analyzed.

## Data Availability

Data are contained within the article.
